# Ecografía cutánea: más allá de la clínica y la dermatoscopia

**DOI:** 10.1016/j.aprim.2024.103062

**Published:** 2024-07-30

**Authors:** Luis Ortiz-González, Basilio Narváez-Moreno, Luis Ortiz-Peces

**Affiliations:** a Departamento de Ciencias Biomédicas, Facultad de Medicina y Ciencias de la Salud, Universidad de Extremadura, Badajoz, España; b Servicio de Cirugía Oral y Maxilofacial, Hospital Universitario La Paz, Madrid, España

El pilomatrixoma, o epitelioma calcificante de Malherbe, es uno de los tumores de tejidos blandos de cabeza y cuello más comunes en la infancia. Se trata de una neoplasia benigna derivada de las células de la matriz del folículo piloso^1^. Representa uno de 800-1.000 de los tumores cutáneos y afecta predominantemente a mujeres. Es más común a una edad temprana, especialmente en las dos primeras décadas de la vida, con un inicio por debajo de los 10 años en el 40% de los casos^2^.

Normalmente se presenta como un nódulo solitario, de crecimiento lento, firme, no doloroso, adherido a la epidermis, pero móvil en el plano subcutáneo y de localización preferente en la cara, el cuello y la extremidad superior proximal^1^.

Esta presentación clínica es tan característica que muchos cirujanos experimentados extirpan los pilomatrixomas sospechosos sin imágenes previas. En cualquier caso, la exéresis quirúrgica sin diagnóstico por imagen previo permitirá un tratamiento correcto en la mayoría de los casos^1^.

Se han documentado imágenes de ultrasonidos, tomografía computarizada y resonancia magnética características del mismo^3^.

Aunque es una lesión bien reconocida, algunos autores cifran entre el 20 y el 50% las tasas de diagnóstico correcto mediante el examen clínico, y próximas al 40% a través del estudio ecográfico^4^, ^5^. Este último, al ser una técnica explorador-dependiente, depende de la tecnología del equipo de ultrasonidos utilizado y del conocimiento, experiencia y habilidad del explorador. De hecho, se ha documentado que la ecografía de alta resolución puede ayudar a confirmar el diagnóstico preoperatorio^1^.

De este modo, las características de ecogenicidad heterogénea, focos ecogénicos internos en un patrón de puntos dispersos y un borde hipoecoico o sombra posterior podrían ser criterios ultrasonográficos discriminativos para diferenciar los pilomatrixomas de otros tumores subcutáneos^6^.

Utilizando transductores de muy alta frecuencia, se han descrito cinco patrones ecográficos que contribuyen al diagnóstico prequirúrgico: tipo I, totalmente calcificado; tipo 2, parcialmente calcificado con halo periférico hipoecoico y avascular; tipo 3, lesión compleja, de bordes no definidos; tipo 4, lesión pseudoquística; tipo 5, pseudoneoplásica, de contornos irregulares y vascularización evidente. Los dos primeros, fácilmente identificables, son las denominadas formas clásicas, en las que se ha documentado un 100% de diagnóstico ultrasónico certero. El más frecuente de ellos es el tipo 2^2^.

Presentamos el caso de una paciente de cinco años que presenta un cuadro de cuatro meses de evolución caracterizado por una lesión nodular, rosada y discretamente dolorosa a la palpación en la piel del dorso nasal, sin mejoría tras antibioterapia tópica.

La clínica y la dermatoscopia (fig. 1) no pudieron determinarla, por lo que se realizó una ecografía con sonda de alta frecuencia en la que se puso de manifiesto una lesión nodular, dérmica, de 3 mm de diámetro, bien delimitada, con halo periférico hipoecogénico, avascular, y ecogenicidad heterogénea central, que interpretamos como puntos incipientemente calcificados en su seno, que no dejan sombra acústica superior por su pequeño tamaño, sugerente de pilomatrixoma tipo 2 de la clasificación de Solivetti et al. (fig. 2).

**Figura 1:**
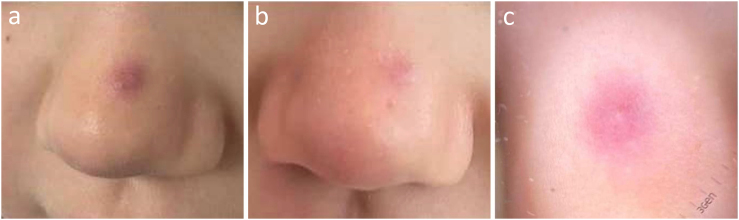
Imagen clínica de la lesión antes del tratamiento (a) y después del tratamiento antibiótico tópico (b). Imagen dermatoscópica de la lesión: eritema difuso, inespecífico, sin patrón melanocítico (c).

**Figura 2:**
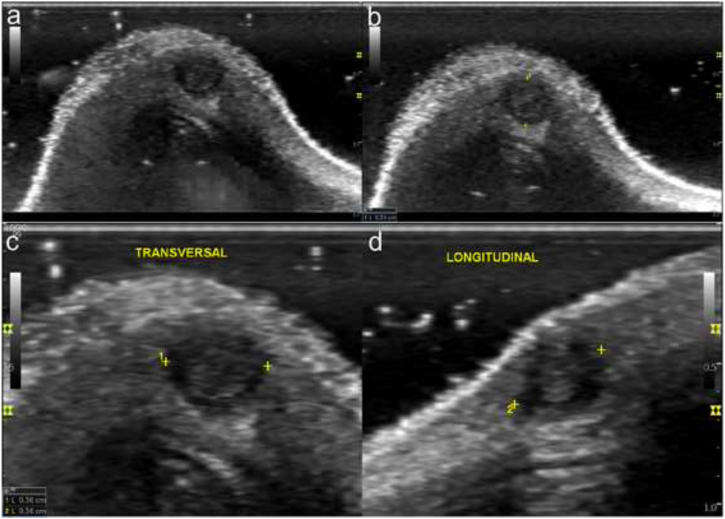
Imágenes ecográficas obtenidas mediante sonda lineal de alta frecuencia (8- 12 MHz). a y b) Cortes transversales del dorso nasal donde se objetiva una lesión nodular, dérmica, de 3 mm de diámetro, bien delimitada, con halo periférico hipoecogénico y puntos calcificados en su seno, correspondiente a un pilomatrixoma tipo II de la clasificación de Solivetti et al.^2^. c y d) Cortes transversal y longitudinal de la misma zona aumentada de tamaño, para una mejor visualización y cuantificación del mismo.

Tras informar a los padres de las características benignas de la lesión, muy sugerente de pilomatrixoma, y dada la localización tan visible de la misma con su posible repercusión estética, se acordó posponer su exéresis y hacer un seguimiento evolutivo del proceso.

En este caso, consideramos que la ecografía ha permitido esclarecer el caso más allá de la clínica y la dermatoscopia, poniendo de manifiesto el tamaño y sus características ultrasonográficas sugerentes de pilomatrixoma, pendiente de la confirmación histológica futura.

## Consideraciones éticas

Los autores confirman que se han obtenido todos los consentimientos requeridos por la legislación vigente para la publicación de cualquier dato personal o imágenes de pacientes, sujetos de investigación u otras personas que aparecen en los materiales enviados a Elsevier, se han realizado todos los procedimientos éticos y se han respetado los derechos de privacidad de los sujetos humanos.

Los autores conservan una copia escrita de todos los consentimientos y, en caso de que Elsevier lo solicite, aceptan proporcionar las copias o pruebas de que de dichos consentimientos han sido obtenidos.

## Financiación

Este trabajo no ha recibido ningún tipo de financiación.

## Conflicto de intereses

Los autores declaran no tener ningún conflicto de intereses.
